# A Crowdsourcing Open Contest to Design a Latino-Specific COVID-19 Campaign: Mixed Methods Analysis

**DOI:** 10.2196/35764

**Published:** 2022-05-12

**Authors:** Harita S Shah, Suzanne Dolwick Grieb, Alejandra Flores-Miller, Katherine H Phillips, Kathleen R Page, Ana Cervantes, Cui Yang

**Affiliations:** 1 Department of Medicine University of Chicago Chicago, IL United States; 2 Department of Pediatrics School of Medicine Johns Hopkins University Baltimore, MD United States; 3 Department of Medicine School of Medicine Johns Hopkins University Baltimore, MD United States; 4 The Esperanza Center Catholic Charities of Baltimore Baltimore, MD United States; 5 Department of Biostatistics Bloomberg School of Public Health Johns Hopkins University Baltimore, MD United States

**Keywords:** crowdsourcing, Latino, open contest, community engagement, social marketing, COVID-19, mixed method, implementation, thematic analysis

## Abstract

**Background:**

Latino communities are among the most heavily impacted populations by the COVID-19 pandemic in the United States due to intersectional barriers to care. Crowdsourcing open contests can be an effective means of community engagement but have not been well studied in Latino populations nor in addressing the COVID-19 pandemic.

**Objective:**

The aims of this study are to (1) implement and evaluate a crowdsourcing open contest to solicit a name for a COVID-19 social marketing campaign for Latino populations in Maryland and (2) conduct a thematic analysis of submitted entries to guide campaign messaging.

**Methods:**

To assess the level of community engagement in this crowdsourcing open contest, we used descriptive statistics to analyze data on entries, votes, and demographic characteristics of participants. The submitted text was analyzed through inductive thematic analysis.

**Results:**

We received 74 entries within a 2-week period. The top 10 entries were chosen by community judges and the winner was decided by popular vote. We received 383 votes within 1 week. The most common themes were collective efficacy, self-efficacy, and perceived benefits of COVID-19 testing. We used these themes to directly inform our social marketing intervention and found that advertisements based on these themes became the highest performing.

**Conclusions:**

Crowdsourcing open contests are an effective means of community engagement and an agile tool for guiding interventions to address COVID-19, including in populations impacted by health care disparities, such as Latino communities. The thematic analysis of contest entries can be a valuable strategy to inform the development of social marketing campaign materials.

## Introduction

Latino communities are among the most heavily impacted populations by the COVID-19 pandemic in the United States, with 1.9 times the infection rate of non-Hispanic White individuals and 2.3 times the age-adjusted mortality rate [1]. Latino individuals account for 18% of the US population but 27.1% of all COVID-19 cases. Among individuals 18-50 years of age, the disparity widens, with Latino adults comprising 42% of COVID-19 cases and 40% of COVID-19 deaths, with mortality rates up to 8 times higher than among non-Hispanic White individuals [2]. Certain subgroups within the diverse Latino populations have been shown to be disproportionately impacted by COVID-19. Latino immigrant populations have experienced a disproportionate burden of COVID-19 infections given occupational exposures, higher household occupancy, and barriers to accessing health care (eg, lack of insurance, fear of deportation, and limited English proficiency) [3-5].

Similar to national trends, the impact of systemic inequities has been painfully evident in our community in Baltimore, Maryland. Baltimore is an emerging immigrant-receiving city that has seen a marked influx of Latino immigrants from Central America over the last 2 decades [6]. Data from Johns Hopkins Health Systems showed that as the pandemic unfolded between March 11, 2020, to May 25, 2020, the overall SARS-CoV-2 positivity rate was 16.3%, but the rate was significantly higher for Latino patients (42.6%) compared to Black (17.6%) and White (8.8%) patients [7]. As the pandemic unfolded, our team partnered with community-based organizations (CBOs) to develop and implement a comprehensive Latino COVID-19 response strategy that addressed systemic barriers to care for Latino populations. The team focused on expanding access to free COVID-19 testing and, later, vaccination through trusted community sites, a bilingual hotline, and a team of bilingual and bicultural peer navigators (or *promotoras*). Although these components were positively received and used by community members, positivity rates remained disproportionately high among Latino populations in Baltimore [8]. Given this disparity and the need to dynamically respond to the evolving COVID-19 pandemic, our team developed and implemented a social marketing intervention as part of the Rapid Acceleration of Diagnostics for Underserved Populations (RADx-UP) initiative [9]. The intervention aim was to use crowdsourcing and a web-based platform to develop culturally congruent messaging to facilitate access to testing as well as expedite linkage to care services and/or *promotora* support.

Crowdsourcing is an effective means of community engagement, based on the principles of collective wisdom and open access [10,11]. Crowdsourcing is defined as “an approach to problem solving which involves an organization having a large group attempt to solve a problem or part of a problem, then sharing solutions” [12]. Open contests are a form of crowdsourcing that allow community members to provide solutions to a problem through a contest; the solutions are then consolidated into a unified product [13,14]. Open contests incorporate local knowledge and culture by directly involving a large number of community members in developing and vetting ideas [15].

The crowdsourcing approach avoids the less effective alternative of top-down interventions and can incorporate community-based participatory research (CBPR) principles by involving community stakeholders throughout the process [16]. In fact, the impetus for this crowdsourcing open contest arose when the name originally proposed in the research grant, *Juntos* (“Together”), was poorly received by community health workers (CHWs) on our team. The CHWs found it reminiscent of political propaganda and military *juntas*; if CHWs did not have such integral roles within our team, we may not have received this important feedback. Thus, we sought community input through this crowdsourcing open contest to develop more representative messaging for the COVID-19 social marketing campaign.

Crowdsourcing methods have been shown to be feasible among Latino populations regardless of English- or Spanish-language preference and thus are suitable to guide our approach to the COVID-19 pandemic [17]. Latino individuals have been shown to have high rates of internet and smartphone use, making web-based outreach a reasonable approach; in 2016, 84% of Latino respondents reported internet and social media use and this use has increased during the COVID-19 pandemic [18,19]. To our knowledge, no studies have been published evaluating crowdsourcing open contests for Latino audiences. The ability to crowdsource virtually makes it an apt tool for efforts to combat COVID-19 given social distancing practices. However, few studies have been published detailing the use or results of crowdsourcing for COVID-19–focused interventions.

To understand and incorporate community members’ attitudes, beliefs, and norms toward the COVID-19 pandemic, we implemented a crowdsourcing open contest to solicit the central messaging for a social marketing campaign promoting equitable COVID-19 testing for Latino populations in Maryland. In this paper, we describe the process and outcomes of a crowdsourcing open contest and thematic analysis of contest entries, which we subsequently used to guide campaign messaging. Prior studies have used the thematic analysis of contest entries to understand community attitudes [20-23]. To our knowledge, this is the first study to use the thematic analysis of contest entries to directly inform intervention development.

## Methods

### Overview

In November 2020, our team implemented a crowdsourcing open contest to name a COVID-19 social marketing campaign for Latino populations in Maryland. The focus of the contest was on COVID-19 testing and general prevention given that vaccines were not publicly available at that time. The contest was guided by CBPR principles in that our team consisted of diverse stakeholders involved in each aspect of the project (eg, CHWs, physicians, researchers, and designers) in addition to the community advisory board (CAB).

**Figure 1:**
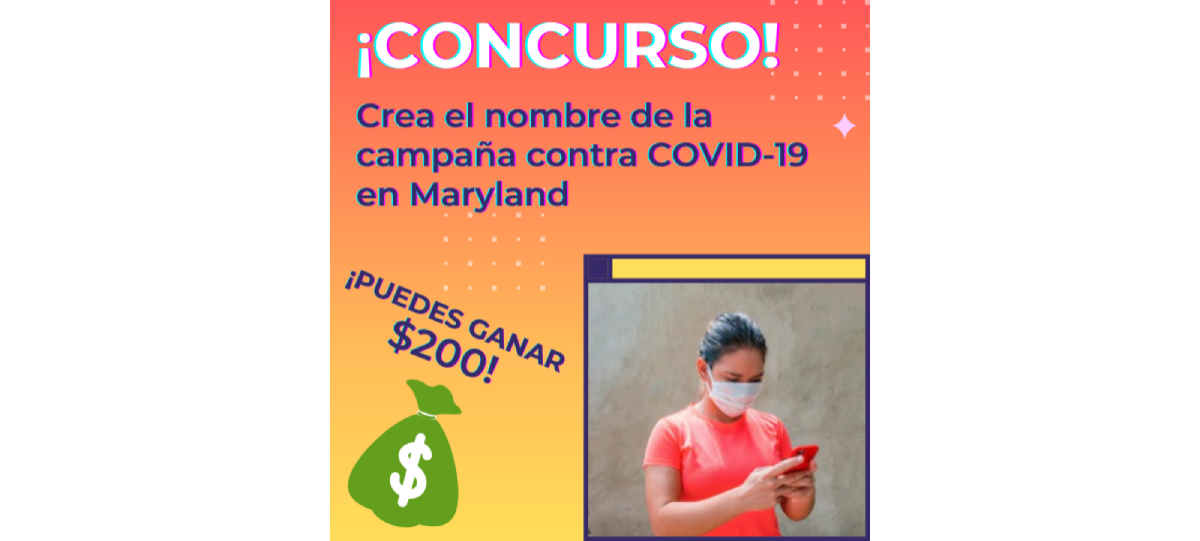
Call for entries for the open contest ("Contest! Name the COVID-19 campaign in Maryland").

Participants were invited to submit a name, hashtag, and an optional call to action (1-2 sentences) for what was referred to as the COVID-19 public health campaign in Maryland. The announcements were written primarily in Spanish with an English translation provided as the target audience for the campaign was Spanish-speaking Latino individuals who may not be reached by city-wide public health campaigns with English messaging (Figure 1).

The implementation and evaluation of the open contest consisted of the following steps: (1) organize a CAB, (2) establish contest rules and incentives, (3) solicit crowd input via community partner social media profiles, (4) evaluate contest entries using both community judge assessments and popular voting, (5) disseminate contest results, and (6) use thematic analysis to understand community values and perspectives so that they may be emphasized in the campaign [20-25].

### Organizing a Community Advisory Board

A CAB was established to represent various CBOs, religious organizations, advocacy groups, and government sectors (ie, the Mayor’s Office on Immigrant Affairs) within Baltimore’s Latino community. Members of the CAB were involved in every stage of this study and the subsequent social marketing intervention. CAB members from nongovernment organizations were compensated with US $50 per meeting. The CAB met monthly to provide guidance on how to promote the open contest to relevant communities, recruit contest participants, translate the winning entry into social marketing messages, and implement the social marketing campaign.

### Establishing Contest Rules and Incentives

The contest was open for entries for 2 weeks (November 13, 2020-November 26, 2020), using a brief timeline given the urgency of the COVID-19 pandemic. Participants submitted a name, hashtag, and an optional call to action (1-2 sentences) via a brief Qualtrics form. Participants were given the option to enter a raffle for 1 of 5 US $25 Visa gift cards by including an email address or phone number. The top 3 entries received US $200, $100, and $50 respectively. At the end of the contest period, 2 study team members independently verified entry eligibility based on the completion of all required fields and relevance to the contest focus of COVID-19.

### Soliciting Crowd Input via Community Partners’ Social Media Profiles

To tailor contest recruitment to Latino immigrants, we announced the contest with Spanish-language posts on community partner social media profiles with established followings, including Somos Baltimore Latino, Centro SOL (Center for Salud/Health and Opportunities for Latinos), Comité Latino, and Sólo Se Vive Una Vez. We primarily focused on CBO Facebook profiles for recruiting given our prior experience with social media reach among Latino immigrants in Baltimore [26]. Our experience and previous studies also showed that complementing web-based activities with in-person recruitment is important for contest inclusivity [23-27]. The contest was advertised in person at our community-based COVID-19 testing site, with a goal to expand the pool to participants who are less engaged in social media.

### Evaluating Contest Entries Using Community Judge Assessments and Popular Voting

Entries were judged based on 3 criteria: creativity, appeal to the local community, and potential for increasing COVID-19 testing and prevention; each criterion was scored on a scale of 1-10. The criteria for judging were shared with all participants at the top of the Qualtrics form. No examples were provided to avoid cognitive fixation, a known obstacle to developing innovative ideas [28-30].

A total of 17 individuals were invited to serve as judges, including 14 Latino community members or leaders, some of whom were also CAB members, and 3 non-Latino individuals who worked in social or health services for the Latino community. A total of 6 judges participated. Judges were provided with a deidentified list of all entries, including the proposed name, hashtag, call to action, and any optional comments submitted. Judges were given 5 days to score all entries in the contest and were compensated US $30 for their time. We totaled the judges’ scores for each entry and used the cumulative score to identify the top 10 entries.

The top entries were shared across the same community partner profiles for a public vote via a Qualtrics form, which was open for 1 week. We notified the participants who submitted the top entries to congratulate them and encourage them to mobilize their social networks to vote. Voters were offered the option to enter a raffle for 1 of 5 US $25 Visa gift cards. At the end of the popular vote, the top 3 finalists were reviewed by the study team to ensure the feasibility of their ideas, meaning they were relevant to the COVID-19 pandemic and there was no overlap with existing campaigns or brands.

### Disseminating Contest Results

The finalists were personally notified and were announced to the public via community partner social media pages, including a weekly live video segment on a popular CBO Facebook page. The winner was also recognized on the campaign website with a photo and a quote sharing their inspiration for participating.

### Mixed-Methods Analysis

To assess the level of community engagement with the crowdsourcing open contest, we measured descriptive statistics for the number of entries and votes and demographic characteristics of the contestants (eg, gender).

The text (campaign name, hashtag, and call to action) was analyzed through inductive thematic analysis by 3 team members. The analysis followed 4 steps: (1) data familiarization, (2) initial coding generation and reduction of codes, (3) development of themes, and (4) development of the hierarchical thematic structure. One team member independently reviewed the text from each entry and developed a formative coding scheme. Then, 2 additional team members reviewed the text and coding scheme, which was revised through discussion among the 3 team members. The codes were then applied to the text, and the resulting coded text was reviewed and discussed by the 3 team members to develop the hierarchical thematic structure [31].

### Application of Themes to the Social Marketing Intervention

We partnered with Altavista Studios, a company with expertise in Spanish-language social marketing campaigns, to design and implement the campaign advertisements. In addition to providing the name of the campaign from the open contest, we also shared the key themes that emerged to guide the content of campaign advertisements. Altavista Studios incorporated these themes in a range of advertisements and provided regular evaluations of advertisement performance through web-based metrics, such as reach and engagement [26,32].

### Ethics Approval

This project was deemed exempt by the Johns Hopkins University Institutional Review Board (CIR00066868).

## Results

### Contest Results

We received 75 submissions in 2 weeks, of which 1 entry was deemed ineligible as it was an advertisement for a business unrelated to the COVID-19 pandemic. Of the 74 eligible entries, 72 (97%) were in Spanish. Over one-quarter (20/74, 27%) of participants were men and 73% (54/74) were women.

The 74 eligible entries were then scored by 6 community judges. With a total possible score range of 18-180, the contest entries ranged from the lowest score of 59 to the highest score of 161. The highest scoring entry was *Descoronando el COVID-19* (“Dethroning COVID-19,” a play on *corona* or “crown”). There was a tie for the 10th best score and thus the top 11 entries were included in the popular vote (see Multimedia Appendix 1).

During the popular vote period for the top entries, we received 383 unique votes within a week. The entry with the most votes was *¡Yo te cuido y tú me cuidas!* (“I take care of you and you take care of me!”). Regrettably, this popular entry was already a name for an established COVID-19 social marketing campaign. A concurrent COVID-19 campaign for a Latino audience with the same name and similar content would confound the evaluation of our intervention, not to mention the potential trademark infringement. We discussed the options with the CAB and collectively decided to announce a tie for first place, with both winners receiving US $200. We also discussed the rationale for selecting the campaign name with the winners for full transparency. The next most popular entry, which was selected to be the name for the campaign, *was Vive Sin Duda* (“Live Without Doubt”).

### Thematic Analysis

A total of 3 themes emerged from the crowdsourcing entries. In Table 1, the themes are presented in order of most to least relevant entries. Although the majority of campaign names related to only 1 theme, many entries touched on more than 1 of the 3 themes through their suggested campaign name, hashtag, and call to action.

**Table 1:** Thematic analysis of crowdsourcing entries.

Themes and subthemes			Number of contest entries^a^		Mean judges’ score (range)^b^	Examples
**Collective efficacy**						
	Community effort	25		90.1 (72-114)		*Juntos contra COVID-19* (“Together against COVID-19”). *Todos unidos* (“Everyone united”).
	Taking care of each other	7		90.6 (59-111)		*Nos cuidamos entre todos* (“We take care of each other”). *¡Yo te cuido y tú me cuidas!* (“I take care of you and you take care of me!”).
**Perceived benefits**						
	Ending COVID-19	11		105.3 (72-161)		*Por un mañana sin COVID-19* (“For a tomorrow without COVID-19”). *Hazte la prueba. Vivamos sin COVID* (“Take the test. Let’s live without COVID”).
	Protecting others	9		83.9 (59-103)		*Hagamos hoy por nuestra familia* (“Let’s do it today for our family”). *Tu salud es primero. Cuidate y cuida a los demás. Haste la prueba* (“Your health is first. Take care of yourself and others. Take the test”).
	Saving life, preventing death	8		81.3 (75-89)		*Salvando vidas* (“Saving lives”). *Ningún contagiado más y menos funerals por COVID-19* (“No more infected and fewer funerals due to COVID-19”).
	Knowing your status	8		92.8 (74-115)		*Vive sin duda* (“Live without doubt”). *¡Siéntete como nueva!* *¡Haste la prueba!* (“Feel like new! Take the test!”).
**Self-Efficacy**						
	N/A^c^	16		95.0 (72-115)		*Luchadores de COVID-19* (“COVID-19 fighters”). *Ama, confía y protégete* (“Love, trust and protect yourself”).

^a^Some entries are counted in more than 1 category. A total of 6 entries were not included under the themes in this table, including the following: El COVID-19 no es un juego (“COVID-19 is not a game”), Pruebas gratis de COVID-19 (“Free COVID-19 tests”).

^b^Total scores for each entry could range from 18 to 180.

^c^N/A: not applicable. There is no subtheme for this category.

### Application of Themes to the Social Marketing Intervention

**Figure 2:**
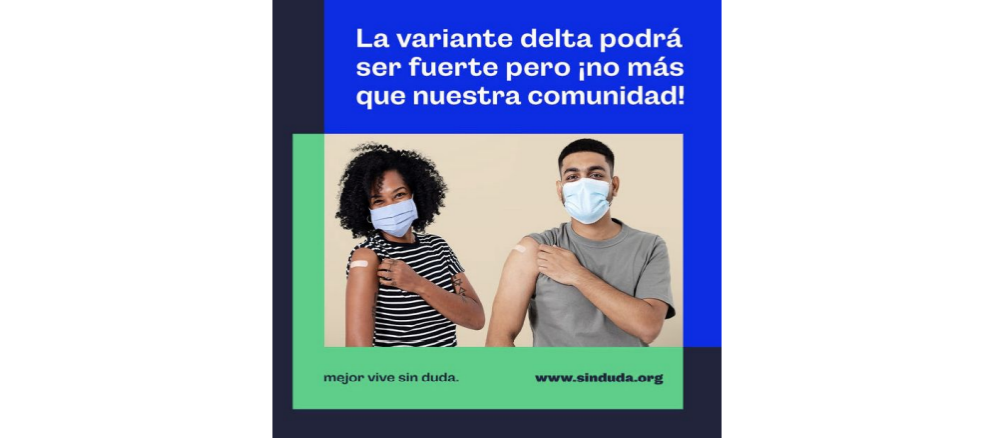
Advertisement aligned with theme of collective efficacy ("The delta variant may be strong, but not as strong as our community!").

**Figure 3:**
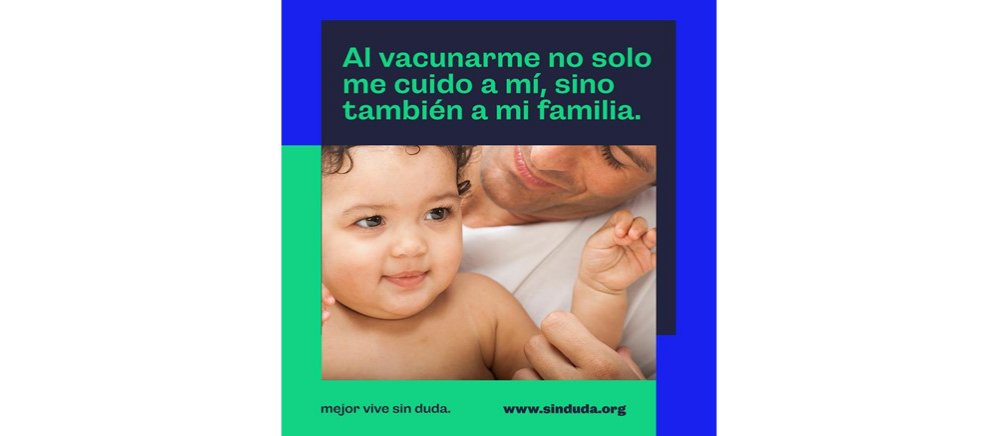
Advertisement aligned with theme of perceived benefits ("By getting vaccinated I'm not only protecting myself, but also my family.").

We shared the 3 themes with our social media partner to develop advertisements that would resonate with the local community. These themes were integrated into the *Vive Sin Duda* (“Live without a doubt”) campaign, hereafter referred to as the *Sin Duda* campaign. Campaign messaging was initially focused on COVID-19 testing but was then adapted to include advertisements on vaccines and COVID-19 variants; we continued to incorporate the 3 themes as messaging topics evolved with the pandemic. Figure 2 provides an example of an advertisement based on the theme of collective efficacy, created in response to the emergence of the Delta variant in fall 2021. An example of messaging based on the theme of perceived benefits with a focus on protecting oneself and one’s family is displayed in Figure 3. In fact, we found that these advertisements were among the highest performing in terms of reach and engagement in comparison to advertisements created independently by the design team.

## Discussion

### Principal Results

Overall, we successfully crowdsourced to guide the messaging for a COVID-19 social marketing campaign for Latinos in Maryland. The winning entry, *Vive Sin Duda*, has since been used for the development of a public health campaign and website [33]. The *Sin Duda* campaign initially focused on COVID-19 testing and, after the approval of the COVID-19 vaccine, subsequently expanded to include vaccination information. This study fills an important gap in the literature as we are the first team to document how to tailor crowdsourcing open contests to Latino populations, especially in response to a public health emergency.

First, in a 2-week period limited by the urgency of the COVID-19 pandemic, we received 74 eligible entries, which were scored by a panel of community judges and a popular vote. The brief duration and specific audience make this project difficult to compare to the existing literature as most published studies were conducted over longer durations of time, with larger populations, and did not focus specifically on Latino populations [16,21]. Compared to contests of similar scope, however, we achieved a similar number of entries [27,34]. We succeeded in soliciting relevant Spanish-language entries from local immigrant community members through targeted CBO recruitment. Given the diversity within Latino populations, it is important to tailor recruitment strategies to reach a specific community through the careful selection of partnerships, platforms, and language. For example, although platforms like Instagram and Tiktok have surpassed Facebook in use among younger populations, our experience has taught us that for Latino immigrant adults in Baltimore, Facebook, and specifically CBO Facebook pages, are most effective [26].

Second, we identified themes that may be useful for future public health campaigns or initiatives that seek to address COVID-19 testing and prevention for Latino populations. The most popular theme, collective efficacy, has been shown to facilitate addressing other health conditions, such as obesity, in Latino populations and has been associated with the potential to reduce health care disparities [35,36]. Another theme that emerged was that of the perceived benefits of COVID-19 testing, mainly the ability to protect oneself and others. The importance of protecting one’s family has emerged as a common driving force among Latino populations, including among young Latino fathers [37].

The final theme that emerged was self-efficacy, defined as an individual’s belief in their ability to plan and execute a course of action [38]. Self-efficacy is a well-known predictor of positive health behaviors across races and ethnicities, including behaviors related to nutrition, physical activity, medication adherence, and HIV testing [39-41]. Recent media coverage of the impact of COVID-19 on Latino populations has often focused on vaccine hesitancy. Our team has found that in addition to addressing intersectional factors that drive vaccine hesitancy, it is important to include strength-based messaging along the theme of self-efficacy, as opposed to deficit-based messaging, while concurrently removing barriers to care [42-44].

Our novel approach of using thematic analysis to directly inform campaign materials resulted in advertisements with high performance and reach. Crowdsourcing open contests offer a low-cost and prompt way to solicit community perspectives and facilitate community-engaged interventions. The findings from this study provide a timely opportunity to document community-driven strategies to make COVID-19 services more accessible and acceptable to marginalized communities.

Given the lack of publications on crowdsourcing open contests focusing on Latino populations, particularly for COVID-19 initiatives, this manuscript provides an important addition to the literature and may guide future crowdsourcing initiatives in similar populations. By building the *Sin Duda* campaign on the foundation of community input, we not only arrived at a name but also elicited key perspectives within the community that helped guide the creation of successful messaging throughout the campaign.

### Limitations

This study has noteworthy limitations. First, although the web-based form facilitated prompt participation and voting, this modality inherently excluded community members who lacked internet access. We selected the web-based modality based on high internet and smartphone use rates among Latino individuals as this is the target audience for the social media campaign [18,19]. However, this contest may have excluded people from more disadvantaged backgrounds who lack internet access or are not engaged with CBOs and are disproportionately impacted by COVID-19. Second, given the short duration of the contest, the number of entries and therefore viewpoints were limited. We tried to optimize community input by using a public vote to choose the winning entries, and we ensured that all campaign messaging incorporated community viewpoints based on input from the CAB and community members obtaining COVID-19 testing. A further limitation is that the entry with the most popular votes was not a feasible choice as it was already in use for another COVID-19 campaign for a Latino audience. We navigated this issue with the guidance of the CAB and thus announced a tie for first place after openly discussing the rationale with the winners. In future contests, all entries should be checked upon submission such that any options that are not original may be disqualified early on to avoid confusion. Lastly, although our findings support the use of crowdsourcing, more rigorous evaluations through randomized controlled trials are needed to compare the effectiveness of crowdsourcing approaches to noncrowdsourcing approaches in campaign development [45,46].

### Conclusions

Crowdsourcing is an effective means of community engagement and an agile tool for guiding interventions to address COVID-19, including in populations affected by health care disparities, such as Latino communities. In considering the role of crowdsourcing in community-engaged research, the thematic analysis of contest entries is a useful and timely way of understanding community values such that they may be prioritized in the content of the intervention.

## Appendix

Multimedia Appendix 1 Top contest entries in order of votes.

## Abbreviations

CAB: community advisory board
CBO: community-based organization
CBPR: community-based participatory research
CHW: community health worker
NIH: National Institutes of Health
RADx-UP: Rapid Acceleration of Diagnostics for Underserved Populations
