# Supplementary material for: A Crowdsourcing Open Contest to Design a Latino-Specific COVID-19 Campaign: Mixed Methods Analysis
Source: JMIR Form Res. 2022 May 12;6(5):e35764. doi: 10.2196/35764 (PMC9106278; doi:10.2196/35764)
Supplement: Multimedia Appendix 1 [file formative_v6i5e35764_app1.docx]

| Campaign Name (Spanish \| *English*) | Judges Score^a^ | Number of Votes  N=383 |
| --- | --- | --- |
| ¡Yo te cuido y tú me cuidas! \| *I take care of you and you take care of me!* | 111 | 107 (27.9%) |
| Vive sin duda. \| *Live without doubt.* | 115 | 62 (16.2%) |
| ¡Chequémonos comunidad! \| *Community, let’s check ourselves!* | 107 | 61 (15.9%) |
| Todos sanando a Maryland \| *Everyone healing Maryland* | 109 | 59 (15.4%) |
| Este virus lo paramos Unidos \| *United we stop this virus* | 114 | 32 (8.4%) |
| Descoronando el COVID-19 \| *Dethroning COVID-19* | 161 | 24 (6.3%) |
| Maryland en acción por nuestra atención \| *Maryland in action for our care* | 107 | 11 (2.9%) |
| Fuera COVID \| *Out of here COVID* | 122 | 10 (2.6%) |
| Sin miedo al COVID \| *Without fear of COVID* | 107 | 9 (2.3%) |
| De esta salimos \| *We come out of this* | 120 | 8 (2.1%) |

Appendix Table. Top Contest Entries in Order of Votes.

^a^ Total scores for each entry could range from 18 to 180.
